# Comparing the Efficacy of Excitatory Transcranial Stimulation Methods Measuring Motor Evoked Potentials

**DOI:** 10.1155/2014/837141

**Published:** 2014-04-03

**Authors:** Vera Moliadze, Georg Fritzsche, Andrea Antal

**Affiliations:** ^1^Department of Clinical Neurophysiology, University Medical Center, Georg-August University of Göttingen, Robert-Koch-Straße 40, 37075 Göttingen, Germany; ^2^Department of Child and Adolescent Psychiatry, Psychosomatics and Psychotherapy, Goethe University of Frankfurt am Main, Deutschordenstraße 50 (House 92), 60528 Frankfurt am Main, Germany

## Abstract

The common aim of transcranial stimulation methods is the induction or alterations of cortical excitability in a controlled way. Significant effects of each individual stimulation method have been published; however, conclusive direct comparisons of many of these methods are rare. The aim of the present study was to compare the efficacy of three widely applied stimulation methods inducing excitability enhancement in the motor cortex: 1 mA anodal transcranial direct current stimulation (atDCS), intermittent theta burst stimulation (iTBS), and 1 mA transcranial random noise stimulation (tRNS) within one subject group. The effect of each stimulation condition was quantified by evaluating motor-evoked-potential amplitudes (MEPs) in a fixed time sequence after stimulation. The analyses confirmed a significant enhancement of the M1 excitability caused by all three types of active stimulations compared to sham stimulation. There was no significant difference between the types of active stimulations, although the time course of the excitatory effects slightly differed. Among the stimulation methods, tRNS resulted in the strongest and atDCS significantly longest MEP increase compared to sham. Different time courses of the applied stimulation methods suggest different underlying mechanisms of action. Better understanding may be useful for better targeting of different transcranial stimulation techniques.

## 1. Introduction

Among electrical stimulation methods, transcranial direct current stimulation (tDCS) is the most well-known noninvasive technique for shaping neuroplasticity in humans [1–5]. Much of the evidence for the neuronal effects of tDCS can be based on physiological studies both in man and in animals [5]. In particular, measuring the amplitude of motor evoked potentials (MEPs) allows easy quantification of excitability changes induced by tDCS, however, only at the primary motor cortex (M1) [2]. Besides tDCS, it was shown that transcranial random noise stimulation (tRNS), in a range either between 0.1 Hz and 640 Hz or between 100 and 640 Hz, is a similarly effective technique for increasing cortical excitability, however, circumventing the directional sensitivity of standard tDCS [6]. Using magnetic stimulation, apart from high frequency repetitive transcranial magnetic stimulation (rTMS), intermittent theta burst stimulation (iTBS) [7] is regarded as the most time-efficient effective technique in order to increase cortical excitability.

So far in all paradigms the duration and the magnitude of the after-effects were controlled by varying stimulation duration, type, or intensity [2, 3, 6, 8–11], nevertheless always in different subjects populations. While the subject-to-subject variability using different methods can be high, the aim of the present study was to compare the efficacy of different transcranial stimulation methods that are supposed to increase cortical excitability, in the same subject population.

## 2. Materials and Methods

### 2.1. Subjects

Twelve subjects (age: 25.7 ± 4.1 years; range: 23–38 years) participated in this study. All subjects were right-handed, according to the short version of the Edinburgh Handedness Inventory, and they were naive with regard to the aim of the study. Those who were ill, were pregnant, were suffering from drug abuse, or had metallic implants/implanted electrical devices were excluded by an interview. None of them took any medication acting on the central nervous system or had a history of a neurological and psychiatric disease. All subjects gave written informed consent. The experimental procedures conformed to the Declaration of Helsinki and were all approved by the Ethics Committee of the University of Göttingen. Subjects and the investigator, who made the MEP measurements, were blinded for stimulation conditions in all of the studies. The stimulations were done by another investigator. All of the subjects were BDNF Val/Val homozygotes [12–16].

### 2.2. Stimulation Techniques

#### 2.2.1. tDCS and tRNS

atDCS and tRNS at 1 mA intensity were delivered by a battery-driven electrical stimulator (Version DC-Stimulator-Plus, NeuroConn GmbH, Ilmenau, Germany) through conductive-rubber electrodes placed in two saline-soaked sponges. With regard to tDCS, only anodal stimulation was introduced. For tRNS, a random level of current was generated for every sample (sampling rate 1280 sps). The random numbers were normally distributed; the probability density function followed a bell-shaped curve. In the frequency spectrum, all coefficients had a similar size. The noise signal contained all frequencies up to half of the sampling rate, that is, a maximum of 640 Hz. The signal had no DC offset. For both atDCS and tRNS, the current was ramped up and down over the first and last 5 s of stimulation. The size of the stimulation electrode over the left M1 was 4 × 4 cm and of the reference electrode 6 × 14 cm, which was placed over the contralateral orbit; both were fixed on the head by elastic bands. The position of the stimulation electrode was determined prior to stimulation by single pulses of transcranial magnetic stimulation (TMS). For sham stimulation, tDCS was turned on for 30 seconds.

#### 2.2.2. iTBS

TBS was applied over the cortical representation field of the right first dorsal interosseous (FDI) muscle and was delivered using a Magstim Super Rapid stimulator. The TBS pattern consisted of bursts containing 3 pulses at 50 Hz repeated at 5 Hz and an intensity of 80% the active motor threshold (AMT). For iTBS, a 2 s train of TBS was repeated every 10 s for a total of 190 s (600 pulses) [17]. AMT was the minimum intensity needed to elicit an MEP response of *∼*200–300 *μ*V during moderate spontaneous background muscle activity (*∼*15% of the maximum muscle strength) in at least three of six consecutive trials.

### 2.3. Measuring Corticospinal Excitability

To examine changes in corticospinal excitability, MEPs of the right FDI were recorded following stimulation of its motor-cortical representation field by single-pulse TMS. These were induced using a Magstim 200 magnetic stimulator (Magstim Company, Whiteland, Wales, UK) with a figure-of-eight standard double magnetic coil (diameter of one winding, 70 mm; peak magnetic field, 2.2 T; average inductance, 16.35 *μ*H). Surface electromyogram (EMG) was recorded from the right FDI through a pair of Ag-AgCl surface electrodes in a belly-tendon montage. Raw signals were amplified, band-pass filtered (2 Hz–2 kHz; sampling rate, 5 kHz), digitized with a micro 1401 AD converter (Cambridge Electronic Design, Cambridge, UK) controlled by Signal Software (Cambridge Electronic Design, version 2.13), and stored on a personal computer for offline analysis. Complete relaxation was controlled through visual feedback of EMG activity, and whenever it was necessary, the subject was instructed to relax. The coil was held tangentially to the skull, with the handle pointing backwards and laterally at 45° from the midline, resulting in a posterior-anterior direction of current flow in the brain. Single-pulse MEPs were recorded with the TMS intensity adjusted to elicit *∼*1 mV peak-to-peak amplitude at baseline. Stimulation intensity was kept constant for the post stimulation assessment. The site was marked with a skin marker to ensure that the coil was held in the correct position throughout the experiment.

### 2.4. Experimental Design

Subjects participated in four different experimental studies. The order of the stimulation conditions with regard to all experiments occurred in a counterbalanced fashion, with at least 5 days between two measurements. Stimulus intensities (in percentage of maximal stimulator output) of TMS were determined at the beginning of each experiment. Immediately following stimulation, 40 single test-pulse MEPs were recorded at 0.25 Hz, that is, approximately 0 min, 5 min, and 10 min after stimulation and then every 10 minutes up to 60 min and then again at 90 min.

### 2.5. Analysis and Statistics

A repeated measure of analysis of variance (ANOVA) (a given current condition versus sham × time points of MEP recordings; dependent variable: mean amplitude of MEPs) was calculated. If a significant main effect of STIMULATION or the interaction of TIME and STIMULATION occurred, a Fisher LSD test was performed. Paired Student's* t*-test was used to compare baseline and poststimulation MEPs. Pearson correlation coefficients were calculated in order to see if there is a correlation with regard to the cortical excitability changes induced by the different stimulation methods. In each subject at every single time point, the effects of two methods (atDCS versus tRNS, atDCS versus iTBS, and tRNS versus iTBS) were pairwise compared. The level of significance was *P* ≤ 0.05.

## 3. Results

No side effects were reported by the subjects after any of the experimental sessions.

The average baseline MEP values were 1.00 ± 0.02 mV for atDCS, 0.96 ± 0.02 for the tRNS condition, 1.01 ± 0.02 for iTBS, and 0.98 ± 0.02 for the sham condition obtained by 44 ± 2.78%, 43.6 ± 2.65%, 44.6 ± 2.85%, and 44 ± 2.86% of maximum stimulator output, respectively (mean ± SEM). Baseline values did not differ between stimulation conditions (*P*s > 0.05). AMT values ranged between 23 and 40% of MSO (mean ± SD 29.7 ± 4.9). There were no correlations between the AMT values and the induced cortical excitability change with regard to any of the stimulation conditions.

As expected, both atDCS and full spectrum tRNS applied over the M1 using 10 min stimulation duration and 1 mA intensity showed the classical behaviour and induced excitability increase. A similar pattern was seen by iTBS.

**Figure 1:**
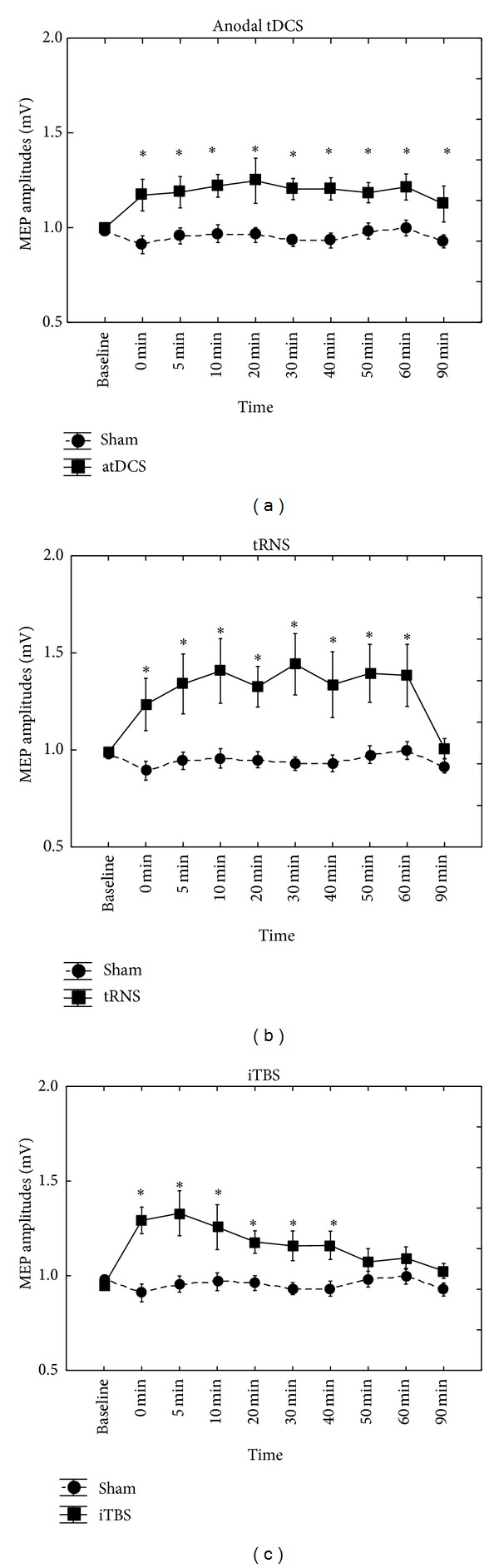
atDCS, full spectrum tRNS applied over the M1 using 10 min stimulation duration and 1 mA intensity showed the classical behaviour and induced excitability increase. Similar pattern was seen by iTBS. Data are mean (± SEM) peak-to-peak amplitudes of MEP. An asterisk indicates that *P* < 0.05.

With regard to atDCS, repeated measurement of ANOVA revealed significant main effects of STIMULATION (*F*_1.11_ = 32.5; *P* = 0.0001). TIME (*F*_9.99_ = 0.83; *P* = 0.6) and the interaction between STIMULATION and TIME were not significant (*F*_9.99_ = 0.96; *P* = 0.5). Fisher LSD test showed significantly higher MEP amplitudes at each time point, compared to sham (*P* < 0.05). Student's* t*-test demonstrated significantly increased MEPs between 10 and 60 minutes compared to baseline (*P* < 0.05), except at 20 minutes (Figure 1).

Concerning tRNS, repeated measures ANOVA revealed significant main effects of STIMULATION (*F*_1.11_ = 10.7; *P* = 0.007) and TIME (*F*_9.99_ = 3.13; *P* = 0.002). The interaction between STIMULATION and TIME was also significant (*F*_9.99_ = 2.99; *P* = 0.003). Fisher LSD test showed significantly higher MEP amplitudes at each time point between 0 and 60 min, compared to sham (*P* < 0.05). Student's* t*-test demonstrated significantly increased MEPs between 5 and 60 minutes compared to baseline (*P* < 0.05) (Figure 1).

**Figure 2:**
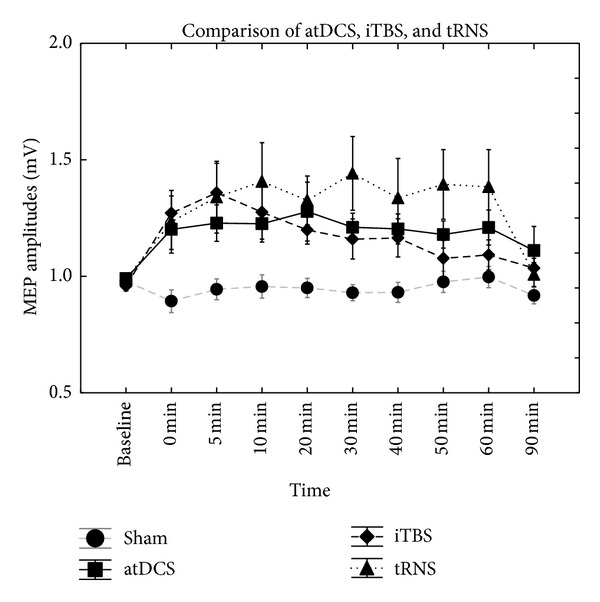
Comparing all of the active stimulation conditions, there were no significant main effects of interactions.

With regard to iTBS, repeated measures ANOVA revealed significant main effects of STIMULATION (*F*_1.11_ = 17.9; *P* = 0.001) and TIME (*F*_9.99_ = 2.57; *P* = 0.01). The interaction between STIMULATION and TIME was also significant (*F*_9.99_ = 2.87; *P* = 0.005). Fisher LSD test showed significantly higher MEP amplitudes between 0 and 40 minutes at each time point, compared to sham (*P* < 0.05). Student's* t*-test demonstrated significantly increased MEPs between 0 and 40 minutes compared to baseline (*P* < 0.05) (Figure 1). Comparing all of the active stimulation conditions, there were no significant main effects of interactions (Table 1, Figure 2). Comparing the three active and one sham stimulation conditions, there was a main effect of STIMULATION (*F*_3.33_ = 6.44; *P* < 0.05).

After the pairwise comparisons of the individual data at every single time point between two methods (atDCS versus tRNS, atDCS versus iTBS, and tRNS versus iTBS), we found no significant correlations between the increases of cortical excitability induced by the different stimulation methods, except between atDCS and iTBS at 10 min after stimulation (Pearson correlation coefficient = 0.71; *P* = 0.005).

## 4. Discussion

The aim of the present study was to compare the efficacy of three widely applied stimulation methods inducing excitability enhancement in the M1. We have used 10 min atDCS, 10 min tRNS at 1 mA intensity, and 3 min iTBS to compare the efficacy of these methods, because for stimulating the M1 these are the most frequently used stimulation durations. Nevertheless, the final effects of noninvasive brain stimulation methods depend on a lengthy list of parameters (e.g., frequency, temporal characteristics, intensity, geometric configuration of the coil/electrode, and current direction), when it is delivered before (offline) or during (online) the task as part of the experimental procedure (for reviews, see, e.g., [5, 18]).

There were significant augmentations of the excitability caused by all three types of active stimulations compared to sham stimulation, with slightly different time courses. tRNS resulted in the strongest (in MEP amplitude increase) and significantly longest MEP increase compared to sham. Nevertheless, between the types of active stimulation conditions there was no significant difference.

On the physiological level (e.g., MEP measurements) or on the behavioural level (e.g., reaction time and performance measures), different transcranial stimulation methods might show comparable effects with regard to the direction and the magnitude of the excitability or behavioral changes [11, 19]. For example, both atDCS and tRNS over M1 resulted in MEP increase using the same stimulation parameters and electrode sizes in a partly overlapping population [20], both applied over the dorsolateral prefrontal cortex (DLPFC) impaired performance in a categorization task [21] in a parallel group design. Nevertheless, opposite results were also observed: tested in the same subjects, atDCS over the left DLPFC improved working memory performance, while tRNS had no effect [22]. Applied over the primary visual cortex, anodal stimulation had no effect on perceptual learning while high frequency tRNS improved it, again in a parallel group design [23]. Similar results were observed in a visuomotor learning task when the stimulation was applied over the M1 [24]. The partly contradictory results are explained by methodological differences (different stimulation parameters with regard to the duration and intensity of the intervention; electrode montage that results in different current flow in the brain; time of the stimulation: during versus before task) and anatomical and physiological differences (variances in the number of excitatory and inhibitory neurons in a given cortical area, orientation of the axons with regard to the current flow, and different concentration of neurotransmitters). Importantly, the results obtained within the motor system are not always equivalent to the results obtained in the visual system or other areas [25, 26]. Furthermore, considering the varying time courses of the applied stimulation methods suggests that the assumption of different mechanisms of actions is likely. Physically, NIBS techniques affect neuronal states through different mechanisms. Changes induced by atDCS are considered to be dependent on the NMDA receptor activity. Long-term tDCS effects are not observed after administration of an NMDA receptor antagonist or blocking Na^+^ channels [27, 28]. The mechanism(s) of tRNS is less clear. tRNS might induce temporal summation of small depolarizing currents, which could interact with the activity of the engaged neurons. Originally, [6] it was suggested that tRNS augments sodium channels activity. Of course, after a short depolarization, repolarization of sodium channels would take time; with repeated stimulation these channels can be reopened with an asymmetrical time course [29]. This seems tantamount to the stochastic resonance phenomenon. tRNS is a noisy stimulation and noise in the nonlinear systems enhances performance through stochastic resonance [30].

In case of anodal tDCS membrane polarization probably induces a sustained depolarization with inactivation of voltage-dependent channels. With regard to the technical application in healthy and clinical populations, tRNS has the advantage of higher cutaneous perception thresholds and lower response rates when compared with atDCS [31].

Concerning iTBS, the mechanism of the pulsatile stimulation might be completely different although pharmacological studies have demonstrated that the after-effects of iTBS at least partially also depend on NMDA receptor activity. The NMDA receptor antagonist memantine blocked the after-effects of iTBS in healthy subjects, whereas placebo did not show a significant effect [32]. Nevertheless, the durations of the after-effects are definitely shorter induced by iTBS, compared to atDCS. Furthermore, there is evidence to suggest that neuroplastic responses to different NBS protocols might be due to activation of different populations of synapses [33].

Both atDCS and tRNS, as well as iTBS, produce cortical excitability changes over time. However, atDCS/tRNS application is distinctly cheaper since it can be performed with a small low-priced battery-driven portable stimulator, also suitable, for example, for home use. Furthermore, since it produces less acoustic noise, skin sensation, like itching or tingling, or muscle twitching, it is more suitable for double-blind, sham-controlled studies and for clinical applications.

In summary, in this study, we compared the magnitude of after-effects of different excitatory transcranial stimulation methods tested in the same healthy subjects and found that tRNS resulted in the strongest and significantly longest MEP increase compared to sham. Between the types of active stimulation, there was no significant difference.

## Figures and Tables

**Table 1:** Results of the repeated measurement ANOVAs.

Stimulation (*I* )	Stimulation (*J* )	Middle difference (*I*-*J* )	SEM	Sig.^a^	95% confidence interval for the difference^a^	
					Upper	Lower
Sham	atDCS	−0.221*	0.039	0.*001 *	−0.345	−0.096
	iTBS	−0.197*	0.047	*0.008 *	−0.346	−0.048
	tRNS	−0.319*	0.098	*0.045 *	−0.633	−0.006

atDCS	Sham	0.221*	0.039	*0.001 *	0.096	0.345
	iTBS	0.024	0.047	1.000	−0.128	0.176
	tRNS	−0.099	0.092	1.000	−0.393	0.195

iTBS	Sham	0.197*	0.047	*0.008 *	0.048	0.346
	atDCS	−0.024	0.047	1.000	−0.176	0.128
	tRNS	−0.123	0.109	1.000	−0.471	0.226

tRNS	Sham	0.319*	0.098	*0.045 *	0.006	0.633
	atDCS	0.099	0.092	1.000	−0.195	0.393
	iTBS	0.123	0.109	1.000	−0.226	0.471

*The middle differencence is on the 0.05-level significance.

^a^Bonferroni corrected sign.
